# Effect of acute hyperglycaemia and/or hyperinsulinaemia on proinflammatory gene expression, cytokine production and neutrophil function in humans

**DOI:** 10.1111/j.1464-5491.2007.02348.x

**Published:** 2008-02

**Authors:** M E Stegenga, S N van der Crabben, M C Dessing, J M Pater, P S van den Pangaart, A F de Vos, M W Tanck, D Roos, H P Sauerwein, T van der Poll

**Affiliations:** * Centre for Infection and Immunity Amsterdam (CINIMA), University of AmsterdamAmsterdam, the Netherlands; † Centre for Experimental and Molecular Medicine, University of AmsterdamAmsterdam, the Netherlands; ‡ Department of Endocrinology and Metabolism, University of AmsterdamAmsterdam, the Netherlands; § Department of Clinical Epidemiology and Biostatistics, University of AmsterdamAmsterdam, the Netherlands; ¶ Sanquin Research, Landsteiner Laboratory, Academic Medical Centre, University of AmsterdamAmsterdam, the Netherlands

**Keywords:** cytokines, hyperglycaemia, hyperinsulinaemia, mRNA, neutrophils

## Abstract

**Aims:**

Type 2 diabetes is frequently associated with infectious complications. Swift activation of leucocytes is important for an adequate immune response. We determined the selective effects of hyperglycaemia and hyperinsulinaemia on lipopolysaccharide (LPS)-induced proinflammatory gene expression and cytokine production in leucocytes and on neutrophil functions.

**Methods:**

Six healthy humans were studied on four occasions for 6 h during: (i) lower insulinaemic euglycaemic clamp, (ii) lower insulinaemic hyperglycaemic clamp, (iii) hyperinsulinaemic euglycaemic clamp, and (iv) hyperinsulinaemic hyperglycaemic clamp. Target levels of plasma glucose were 12.0 mmol/l (hyperglycaemic clamps) or 5.0 mmol/l (euglycaemic clamps). Target plasma insulin levels were 400 pmol/l (hyperinsulinaemic clamps) or 100 pmol/l (lower insulinaemic clamps).

**Results:**

Hyperglycaemia reduced LPS-induced mRNA expression of nuclear factor of κ light polypeptide gene enhancer in B cells inhibitor alpha (*NFKBIA*), interleukin-1 alpha (*IL1A*) and chemokine (C-C motif) ligand 3 (*CCL3*), whereas during hyperinsulinaemia enhanced mRNA levels occurred in six out of eight measured inflammation-related genes, irrespective of plasma glucose levels. Combined hyperglycaemia and hyperinsulinaemia led to enhanced *IL1A*, interleukin-1 beta (*IL1B*) and *CCL3* mRNA levels upon LPS stimulation. Neither hyperglycaemia nor hyperinsulinaemia altered cytokine protein production, neutrophil migration, phagocytic capacity or oxidative burst activity.

**Conclusions:**

These results suggest that short-term hyperglycaemia and hyperinsulinaemia influence the expression of several inflammatory genes in an opposite direction, that the acute effects of hyperinsulinaemia on inflammatory mRNA levels may be stronger than those of hyperglycaemia, and that the effects of insulin, in particular, may be relevant in the concurrent presence of hyperglycaemia.

Diabet. Med. 25, 157–164 (2008)

## Introduction

Infection is a common and serious complication of diabetes mellitus and a well-recognized cause of morbidity and mortality [1–3]. It has been estimated that infections account for up to 22% of deaths of patients with diabetes. One explanation for this increased sensitivity to infections may be an impaired innate immune response in diabetic patients. Both elevated plasma glucose and insulin, common features of Type 2 diabetes, influence the immune system [4,5]. Neutrophils play an important role during the early host response to infection by a coordinated series of effector functions that include chemotaxis, phagocytosis and the generation of reactive oxygen species (respiratory burst). Neutrophil chemotaxis is hampered in diabetic patients [6,7]. In one study, hyperglycaemia accounted for this impairment [8], although another study has shown no correlation between chemotaxis and glycaemic levels in diabetic patients [9]. In addition, neutrophil phagocytic capacity of diabetic patients was reduced in some [7,10] but not all investigations [9,11]. Further, all studies [12–16] except one [17] have shown that hyperglycaemia decreases the respiratory burst capacity of neutrophils.

In addition to neutrophil functions, the production of cytokines may also be altered by the presence of hyperglycaemia and/or hyperinsulinaemia. Diabetic patients have displayed elevated resting levels of tumour necrosis factor (TNF)-α, interleukin (IL)-6 and IL-8 [18–20]; however, effects of glucose and insulin on lipopolysaccharide (LPS)-induced proinflammatory cytokine responses *in vitro* and *in vivo* are contradictory [21–25]. Hyperglycaemia up-regulated the expression of several proinflammatory genes, including those encoding for IL-1β and TNF-α*in vitro* [26], the latter gene via increased recruitment of nuclear factor kappa B (NF-κB) p65 to the TNF-α promoter. These results have been confirmed in a porcine model of diabetes [27].

Studies of the effect of hyperglycaemia and hyperinsulinaemia on neutrophil functions and proinflammatory gene expression in Type 2 diabetic patients do not provide insight into the distinct effects of elevated glucose and insulin concentrations, since both are present at the same time. Moreover, diabetic patients almost invariably have other metabolic disturbances such as dyslipidaemia and increased levels of advanced glycation end-products. In addition, to our knowledge no studies in humans have investigated separate hyperglycaemia without hyperinsulinaemia, as experimental glucose infusion will induce rapid production of insulin. Therefore, we sought to determine the distinct effects of short-term hyperinsulinaemia and/or hyperglycaemia on inflammatory mRNA levels and neutrophil functions in healthy human volunteers.

## Methods

### Subjects and design

The present study was performed by an investigation that determined simultaneously the effects of short-term hyperglycaemia and/or hyperinsulinaemia on coagulation and fibrinolysis. The results of that previous study have been published elsewhere [28]. In brief, six healthy, non-smoking, male volunteers [age (mean ± sem) 21.7 ± 0.5 years; weight 73.2 ± 2.0 kg; body mass index 21.8 ± 0.4 kg/m^2^] were studied. All volunteers had normal plasma values of fasting glucose and insulin, and all had a normal oral glucose tolerance test. The study was approved by the Medical Ethical Committee of the Academic Medical Centre in Amsterdam and all subjects gave written informed consent. The study had a cross-over design, with a wash-out period of 4 weeks, and was done in balanced assignment. Each volunteer served as his own control and was studied on four occasions, during a lower insulinaemic euglycaemic (L_insu_E_gluc_) clamp (target insulin level 100 pmol/l, target glucose level 5.0 mmol/l), a lower insulinaemic hyperglycaemic (L_insu_H_gluc_) clamp (insulin 100 pmol/l, glucose 12.0 mmol/l), a hyperinsulinaemic euglycaemic (H_insu_E_gluc_) clamp (insulin 400 pmol/l, glucose 5.0 mmol/l) and a hyperinsulinaemic hyperglycaemic (H_insu_H_gluc_) clamp (insulin 400 pmol/l, glucose 12.0 mmol/l). After an overnight fast the subjects were admitted to the clinical research unit and confined to bed. At 08.45 h a catheter was placed into an antecubital vein for infusion of insulin, somatostatin, glucagon and glucose 10 or 20%. Another catheter was inserted retrogradely into a contralateral hand vein kept in a thermoregulated (60°C) Plexiglas box for sampling of arterialized venous blood. At *T* = 0 (09.00 h), infusions of somatostatin (250 µg/h; Somatostatine-ucb; UCB Pharma BV, Breda, the Netherlands) to suppress endogenous insulin and glucagon secretion, and glucagon (1 ng kg^−1^ min^−1^; Glucagen, Novo Nordisk, Alphen a/d Rijn, the Netherlands) to replace endogenous glucagon concentrations were started; concurrently infusions of insulin (Actrapid; Novo Nordisk) at a rate of 10 or 40 mU/m^2^ body surface area per min (lower or hyperinsulinaemic clamp, respectively) and glucose 10 or 20% at a variable rate to obtain eu- or hyperglycaemia were started. Glucose 20% was used during the L_insu_E_gluc_ clamp; in the other clamps glucose 10% was used to prevent the possibility of phlebitis induced by the high infusion rates required. All infusions were administered by calibrated syringe pumps (Perfusor fm; Braun, Melsungen AG, Germany). To clamp glucose at 5.0 or 12.0 mmol/l (eu- or hyperglycaemic) from *T* = 0:00 until *T* = 6:00, every 5 min the bedside plasma glucose concentration was measured on a Beckman glucose analyser 2 (Beckman, Palo Alto, CA, USA). At *T* = 0:00, *T* = 3:00 and from *T* = 5:40 until *T* = 6:00 every 10 min blood samples were drawn for determination of the concentration of plasma insulin.

### Blood collection

Blood was collected directly before the initiation of the infusions (*T* = 0) and at the end of the infusions (*T* = 6). Leucocyte counts and differentials were determined in ethylenediamine tetraaceticacid (EDTA)-anticoagulated blood using a Stekker analyser (counter STKS; Coulter Counter, High Wycombe, UK). Heparin-anticoagulated blood was used for all other assays. Plasma insulin concentrations were determined with a chemiluminescent immunometric assay (Immulite 2000; Diagnostic Products Corp., Los Angeles, CA, USA).

### Whole blood stimulation and determination of mRNA expression

In order to study whole-blood mRNA levels of inflammatory genes, we chose to do so after LPS stimulation, because non-stimulated whole blood yields very low or undetectable levels of mRNA for inflammatory genes (own unpublished observations). Heparinized whole blood (1 ml) was diluted with an equal volume of RPMI-1640 (GibcoBRL, Invitrogen, Breda, the Netherlands) or RPMI-1640 containing LPS (from *Escherichia coli* O111:B4; Sigma, St Louis, MO, USA) in a final concentration of 10 ng/ml and incubated in polypropylene tubes for 2 h at 37°C. RNA isolation and the mRNA expression of nuclear factor of kappa light polypeptide gene enhancer in B cells 1 (p105; gene symbol *NFKB1*), nuclear factor of kappa light polypeptide gene enhancer in B cells inhibitor, alpha (*NFKBIA*), IL-1 alpha (*IL1A*), IL-1 beta (*IL1B*), TNF-α (*TNF*), IL-6 (*IL6*), IL-8 (*IL8*) and chemokine (C-C motif) ligand 3 (*CCL3*) were determined using the multiplex ligation-dependent probe amplification method exactly as described previously [29–31]. All samples were tested with the same batch of reagents. The levels of mRNA for each gene were expressed as a normalized ratio of the peak area divided by the peak area of the β_2_-microglobulin gene (*B2M*), resulting in relative abundance of mRNAs of the genes of interest [29–31].

### Cytokine production

Whole blood was stimulated exactly as described above, except that all samples were stimulated for 24 h. Protein concentrations of TNF-α, IL-1β, IL-6 and IL-8 were measured in supernatants by cytometric beads array multiplex assay (BD Biosciences, San Jose, CA, USA).

### Neutrophil migration assay

Erythrocytes of heparinized whole blood were lysed with an ice-cold lysis buffer containing 155 mm NH_4_Cl, 10 mm KHCO_3_ and 0.1 mm EDTA (pH 7.4). Leucocytes were resuspended at 5 × 10^6^/ml, incubated with 4 µg/ml Calcein-AM (Molecular Probes, Eugene, OR, USA) for 30 min at 37°C and washed with phosphate-buffered saline (PBS), after which they were resuspended at 2 × 10^6^/ml. The chemoattractants complement factor 5a (C5a) and platelet-activating factor (PAF; both from Sigma) were both prepared in HEPES buffer at concentrations of 10 nm and 100 nm, respectively. A 24-well plate (Multiwell; Becton Dickinson, Le Pont De Claix, France) was prefilled with 800 µl of these chemoattractant solutions or HEPES buffer without chemoattractant. One well was filled with 800 µl of cell suspension. The plate was preheated at 37°C for 5 min. Transwell inserts with a pore size of 3.0 µm (HTS Fluoroblok; Becton Dickinson) were filled with 300 µl of calcein-labelled cells and placed into the Multiwell plate. The plate was placed into a Cytofluor 4000 reader (Applied Biosystems, Foster City, CA, USA). Measurements were performed in triplicate every 2 min for 46 min, using excitation/emission wavelengths of 485/530 nm. The end-point was defined as the total cell migration after 46 min and was calculated as the total extent of fluorescence increase. We also calculated the maximal migration velocity per min by dividing the maximal increase in fluorescence observed between two measurements by two. Both measurements were corrected for the mean fluorescence of 800 µl of cell suspension, and for the percentage of neutrophilic granulocytes in the whole blood of the volunteer, and were calculated either as relative fluorescence units (RFU), or as delta RFU per min (dRFU/min).

### Respiratory burst assay

Nicotinamide adenine dinucleotide phosphate-oxidase activity was assessed as hydrogen peroxide release determined by an Amplex Red kit (Molecular Probes). Neutrophils (0.25 × 10^6^/ml) were stimulated with buffer or with 100 ng/ml phorbol 12-myristate 13-acetate (Sigma), in the presence of Amplex Red (0.5 µm) and horseradish peroxidase (1 U/ml), in a black 96-well plate with a clear bottom (Greiner, Alphen a/d Rijn, the Netherlands). Fluorescence was measured with a Cytofluor 4000 platereader (Applied Biosystems) using excitation/emission wavelengths of 530/580 nm. Measurements were performed in triplicate every min for 35 min. The results were calculated as the maximal increase in fluorescence per min. The results were corrected for the percentage of neutrophilic granulocytes in the whole blood of the volunteer and for the negative control, and depicted as increase of RFU per min (dRFU/min).

### Phagocytosis

The uptake of *E. coli* by neutrophils was analysed essentially as described previously [32,33]. Heat-killed *E. coli* were labelled with fluorescein isothiocyanate (Sigma-Aldrich, St Louis, MO, USA) and added to 100 µl of heparinized whole blood (bacterium/neutrophil ratio of 25 : 1). Bacteria and cells were incubated for 12 min at 37°C and also at 4°C as a negative control. Phagocytosis was stopped by immediately transferring the cells to 4°C and washing them with ice-cold FACS buffer (PBS supplemented with 0.01% NaN_3_, 0.5% bovine serum albumin and 0.35 mm EDTA). The cells were treated with vital blue stain (Orpegen, Heidelberg, Germany) to quench extracellular fluorescence, washed with FACS buffer and analysed using a FACS Calibur flow cytometer (Becton Dickinson, Mountain View, CA, USA). Neutrophils were gated based on forward and side scatter. Results are expressed as phagocytosis index, defined as the percentage of cells with internalized *E. coli* times the mean fluorescence intensity (corrected for the negative controls).

### Statistical analysis

To analyse the effect of hyperinsulinaemia and/or hyperglycaemia and their interaction, results of the four clamps were compared using a repeated measures analysis of variance (repeated covariance type: compound symmetry). Data were checked for normal distribution and equal variances of the residuals. Depending on the results of these tests, data were analysed either parametrically or non-parametrically (rank-transformed data) [34]. Results are presented as percentage change relative to *T* = 0 as mean (± sem). Probability values of < 0.05 were considered statistically significant. Probability values of overall insulin, glucose and interaction effects are stated as *P*_insu_, *P*_gluc_ and *P*_interaction_, respectively. SPSS statistical software version 12.0.1 (SPSS Inc., Chicago, IL, USA) was used to analyse the data.

## Results

### Glucose and insulin

Glucose and insulin levels during the four clamps have been reported previously [28].

### White blood cell count and differentiation

During all clamps a small but statistically significant decrease in total leucocyte counts occurred, which was primarily caused by a small decrease in neutrophil counts (*P* < 0.05 in all four clamps, Table 1). Importantly, leucocyte counts and differentials did not differ between the four clamps, with the exception of lymphocyte counts, which were slightly but statistically significantly lower at the end of the H_insu_H_gluc_ clamp.

**Table 1:** Leucocyte counts and differentials

	L_insu_E_gluc_		H_insu_E_gluc_		L_insu_H_gluc_		H_insu_H_gluc_	
				
Cell subset (10^6^/ml)	*T* = 0	*T* = 6	*T* = 0	*T* = 6	*T* = 0	*T* = 6	*T* = 0	*T* = 6
Leucocytes	5.3 ± 0.6	4.2 ± 0.3*	4.9 ± 0.3	4.0 ± 0.2*	6.0 ± 0.5	4.8 ± 0.4*	5.3 ± 0.5	4.0 ± 0.5*
Neutrophils	2.9 ± 0.6	1.8 ± 0.2*	2.5 ± 0.3	1.8 ± 0.2*	3.5 ± 0.4	2.4 ± 0.2*	2.9 ± 0.3	2.0 ± 0.4*
Lymphocytes	1.7 ± 0.2	1.8 ± 0.2	1.7 ± 0.2	1.6 ± 0.1	1.7 ± 0.2	1.7 ± 0.2	1.7 ± 0.1	1.5 ± 0.1*,**
Monocytes	0.4 ± 0.0	0.4 ± 0.0	0.4 ± 0.0	0.4 ± 0.0	0.5 ± 0.1	0.4 ± 0.0	0.5 ± 0.1	0.4 ± 0.1*

* *P* < 0.05 vs. baseline of clamp;

** *P* < 0.05 vs. L_insu_E_gluc_ clamp.

Absolute leucocyte and subset counts at *T* = 0 and *T* = 6 h, i.e. the time points at which proinflammatory gene expression and neutrophil functions were investigated. Results are shown as mean ± sem.

### Proinflammatory gene expression

**FIGURE 1:**
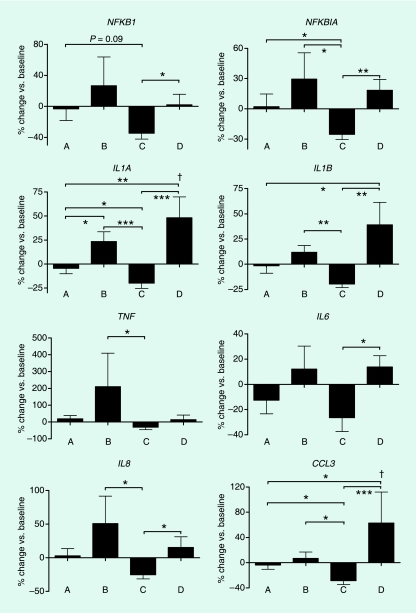
Influence of hyperinsulinaemia and/or hyperglycaemia on proinflammatory mRNA levels. Six subjects were studied on four separate occasions: during a lower insulinaemic euglycaemic (L_insu_E_gluc_) clamp (A), a hyperinsulinaemic euglycaemic (H_insu_E_gluc_) clamp (B), a lower insulinaemic hyperglycaemic (L_insu_H_gluc_) clamp (C) and a hyperinsulinaemic hyperglycaemic (H_insu_H_gluc_) clamp (D). Whole blood obtained at *T* = 0 and *T* = 6 h was stimulated for 2 h with lipopolysaccharide. White blood cells were analysed for mRNA levels relative to mRNA levels of the *B2M* household gene. Data are the mean (± sem) changes in mRNA level ratios at the end of the clamps relative to the change detected at baseline. *P < 0.05; **P < 0.01 and ***P < 0.001; †*P* < 0.05 for interaction of hyperglycaemia and hyperinsulinaemia.

Whole blood that was incubated with control medium yielded very low or undetectable mRNA levels at both *T* = 0 and 6 h (data not shown). As expected, LPS stimulation of blood leucocytes of all subjects at *T* = 0 h (Table 2) and 6 h (data not shown) induced strong up-regulation of *NFKB1*, *NFKBIA*, *IL1A*, *IL1B*, *TNF*, *IL6*, *IL8* and *CCL3* expression. To determine the effect of hyperglycaemia and/or hyperinsulinaemia on leucocyte responsiveness to LPS, we expressed the extent of LPS-induced up-regulation of various genes at *T* = 6 h as a percentage of the extent of LPS-induced up-regulation at *T* = 0 h for each individual. Considering that each volunteer was studied in all four clamps, the changes in LPS-induced gene expression as measured at the end of each clamp (*T* = 6 h) were then compared for each individual (Fig. 1). The extent of proinflammatory gene expression did not change during the L_insu_E_gluc_ clamp. Hyperglycaemia and hyperinsulinaemia had distinct effects on LPS-induced proinflammatory gene expression. Hyperglycaemia *per se* (in the absence of hyperinsulinaemia) in general resulted in attenuated LPS-induced gene expression when compared with the other three clamps; in particular, the expression of *NFKB1* (*P* = 0.09), *NFKBIA*, *IL1A* and *CCL3* (all *P* < 0.05) decreased when compared with the L_insu_E_gluc_ clamp. In contrast, hyperinsulinaemia enhanced LPS-induced gene expression of several cytokine genes, and its effect on mRNA induction of *IL1A*, *IL1B* and *CCL3* was especially clear in the concurrent presence of hyperglycaemia. To check for an overall insulin effect, LPS-induced gene expression at the end of the lower insulinaemic clamps was compared with gene expression at the end of both high insulinaemic clamps. Hyperinsulinaemia in either the presence or the absence of hyperglycaemia induced enhanced mRNA levels of all measured genes (*P*_insu_ < 0.05), except for *NFKB1* (*P*_insu_ = 0.09) and *TNF* (*P*_insu_ = 0.16). Thus, hyperinsulinaemia enhanced levels of several inflammation-related genes, and this effect was irrespective of simultaneous glucose levels for *NFKBIA*, *IL1B*, *IL6* and *IL8*. We did not find an overall glucose effect on LPS-stimulated whole blood mRNA (*P*_gluc_ > 0.05 for all measured genes). Finally, there was a significant interaction between hyperglycaemia and hyperinsulinaemia on post-clamp mRNA levels of *IL1A* and *CCL3* (*P*_interaction_ < 0.05).

**Table 2:** LPS-induced inflammatory gene expression in blood leucocytes

		Expression (fold over *B2M*)	
		
mRNA	Genbank	Med	LPS
*NFKB1*	M58603	0.19 ± 0.01	0.77 ± 0.07*
*NFKBIA*	NM_020529	0.70 ± 0.09	2.34 ± 0.16*
*IL1A*	X02851	0.30 ± 0.01	1.64 ± 0.10*
*IL1B*	M15330	0.51 ± 0.12	4.78 ± 0.19*
*TNF*	M10988	0.06 ± 0.01	0.51 ± 0.06*
*IL6*	M14584	0.03 ± 0.01	2.39 ± 0.20*
*IL8*	M17017	0.61 ± 0.07	2.98 ± 0.25*
*CCL3*	NM_002983	0.33 ± 0.09	5.97 ± 0.35*

Influence of lipopolysaccharide (LPS) stimulation on whole blood mRNA expression profiles. Whole blood drawn from untreated volunteers at *T* = 0 h was incubated for 2 h with either medium control (Med) or LPS. mRNA profiles of all samples were determined with the multiplex ligation-dependent probe amplification method. Values represent relative expression of indicated mRNA levels compared with mRNA levels of β_2_-microglobulin. Results are shown as mean ± sem.

* *P* < 0.0001 vs. negative control. For samples that were below detection level (only present in the medium-stimulated group), the detection level was used in calculations. Differences between medium and LPS stimulation are therefore underestimated.

### Cytokine expression

In a similar way as mRNA expression was analysed, we investigated changes in LPS-induced production of TNF-α, IL-1β, IL-6 and IL-8 as measured at *T* = 6 h, for each individual (data not shown). There was no difference in cytokine production between any of the four separate clamps (*P* > 0.05 for all cytokines). In addition, we observed no overall effect of insulin, glucose or interaction of either condition on cytokine levels (*P*_insu_, *P*_gluc_ and *P*_interaction_ > 0.05 for all cytokines).

### Neutrophil functions

**FIGURE 2:**
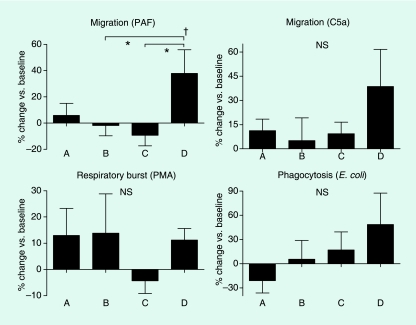
Influence of hyperinsulinaemia and/or hyperglycaemia on neutrophil migration, respiratory burst and phagocytic capacity. Six subjects were studied on four separate occasions: during a lower insulinaemic euglycaemic (L_insu_E_gluc_) clamp (A), a hyperinsulinaemic euglycaemic (H_insu_E_gluc_) clamp (B), a lower insulinaemic hyperglycaemic (L_insu_H_gluc_) clamp (C) and a hyperinsulinaemic hyperglycaemic (H_insu_H_gluc_) clamp (D). Upper panels: neutrophil migration toward platelet-activating factor and complement 5a. Lower left panel: respiratory burst induced by phorbol 12-myristate 13-acetate. Lower right panel: phagocytosis of *Escherichia coli*. Data are the mean (± sem) values at the end of the clamps relative to the values measured at baseline. *P < 0.05; †*P* < 0.05 for interaction of hyperglycaemia and hyperinsulinaemia.

We studied three neutrophil functions considered important for adequate innate immune response to invading bacteria: migration, respiratory burst and phagocytosis (Fig. 2). All measurements performed at *T* = 6 h were expressed as a percentage of the values measured at *T* = 0 h, and the changes in neutrophil functions as measured at the end of each clamp (*T* = 6 h) were compared for each individual in the four different clamps. Neither hyperglycaemia nor hyperinsulinaemia *per se* influenced neutrophil functions. The only effect measured was increased neutrophil migration toward PAF at the end of the H_insu_H_gluc_ clamp, when compared with the clamps with hyperglycaemia or hyperinsulinaemia alone. This effect was not seen when C5a was used as chemoattractant.

## Discussion

In this study we have investigated the acute influence of hyperglycaemia and/or hyperinsulinaemia, using 6-h clamps in healthy humans, on mRNA levels of inflammation-related genes and neutrophil function assays. In this strictly controlled setting, in which each subject served as his own control in four different conditions, hyperglycaemia led to slightly decreased LPS-stimulated mRNA levels of *NFKBIA*, *IL1A* and *CCL3* compared with euglycaemia, whereas hyperinsulinaemia caused increased mRNA levels in the majority of measured inflammatory genes. Relative to the effect of hyperinsulinaemia alone, combined hyperglycaemia and hyperinsulinaemia led to a further increase in *IL1A*, *IL1B* and *CCL3* mRNA levels. Six hours of hyperinsulinaemia and/or hyperglycaemia had no significant effects on cytokine protein production or on crucial neutrophil functions, although the combined presence of hyperinsulinaemia and hyperglycaemia enhanced neutrophil migration when PAF was used as chemoattractant.

The number of studies investigating the role of hyperglycaemia or hyperinsulinaemia on inflammatory gene expression is limited. In monocyte-like THP-1 cells, 72 h of hyperglycaemia induced gene expression of several cytokines, chemokines and adhesion molecules [26]. In the same cell line and in human monocyte-derived macrophages, supraphysiological insulin concentrations stimulated the production of TNF-α mRNA and protein [22]. Also in cultured human myotubes, supraphysiological doses of insulin induced an inflammatory transcriptional response [35]. These last three studies have examined the direct effects of either hyperglycaemia or hyperinsulinaemia, and reported (with the exception of two out of 41 measured genes [26]) small to moderate increases in proinflammatory gene expression profiles (from 1.5 to 5 times gene expression compared with control). Our observation that hyperglycaemia induced lower mRNA levels of *NFKBIA*, which encodes the NF-κB-inhibitor protein ‘inhibitor of kappa-Bα’ (IκBα), is in line with observations that IκBα protein levels decreased in healthy humans after an oral glucose load, although in this last study insulin levels were not suppressed by somatostatin [36]. Despite this, mRNA levels of the proinflammatory genes *IL1A* and *CCL3* were also lower, and *NFKB1* was also not up-regulated after hyperglycaemia. We do not have an explanation for this apparent distinction. However, the observed changes in mRNA level were not accompanied by any change in concentrations of cytokine proteins. The induction of acute hyperglycaemia in healthy humans elevated circulating levels of TNF-α and IL-6 [37], although these elevations were very modest, especially when compared with cytokine increases observed during inflammatory reactions. Plasma protein levels of TNF-α and IL-6 in Type 2 diabetic patients are only moderately higher (1.5–2 times the concentration of non-diabetic control subjects) [38,39]. To examine the effects of hyperglycaemia and/or hyperinsulinaemia after a proinflammatory stimulus, we studied gene expression profiles in unfractionated blood leucocytes during LPS stimulation. We intentionally chose to examine whole blood leucocytes in order to keep the cells in their hyperglycaemic and/or hyperinsulinaemic environment during stimulation and to avoid artificial gene expression due to procedures to isolate specific cell types. Given that leucocyte counts were not influenced by the presence of hyperglycaemia and/or hyperinsulinaemia, it is valid to compare LPS-induced mRNA expression in the different clamps. Our results show that 6 h of hyperglycaemia and/or hyperinsulinaemia lead to changes in LPS-induced expression of some but not all inflammatory genes evaluated. Remarkably, hyperglycaemia seemed to reduce gene expression (significantly so for *NFKBIA*, *IL1A* and *CCL3*), whereas hyperinsulinaemia enhanced the expression of six out of eight measured inflammatory genes. The hyperinsulinaemic effects on gene expression occurred irrespective of simultaneous plasma glucose concentrations, except for *IL1A* and *CCL3*. This remarkable finding implicates that in our tightly controlled setting, the effects of hyperinsulinaemia overrule those observed in hyperglycaemia alone. At the same time, hyperinsulinaemia enhanced expression of some of these genes, particularly in the presence of hyperglycaemia. These results suggest that: short-term hyperglycaemia and hyperinsulinaemia influence the expression of several inflammatory genes in an opposite direction; the acute effect of hyperinsulinaemia may be more powerful compared with hyperglycaemia *in vivo* on inflammatory mRNA levels; and that the effects of insulin, in particular, may be relevant in the concurrent presence of hyperglycaemia. Recently, two studies have examined the effects of hyperinsulinaemia on LPS-induced cytokine release in healthy humans *in vivo* [40,41]. In both studies hyperinsulinaemia modestly increased LPS-induced IL-6 release while not influencing TNF-α levels. Other cytokines were not evaluated in these studies. Of note, the insulin levels achieved in these studies were much higher (800–1200 pmol/l) than in our current investigation (around 400 pmol/l).

Type 2 diabetes has been associated with a number of neutrophil dysfunctions. Most (albeit not all) investigations in Type 2 diabetic patients have reported reduced neutrophil migration, phagocytic capacity and respiratory burst [4,7,9–11]. Our findings strongly argue against an acute effect of hyperglycaemia and/or hyperinsulinaemia on these neutrophil functions, which are considered important for antibacterial defence. Indeed, neither hyperglycaemia nor hyperinsulinaemia influenced neutrophil migration, respiratory burst activity or phagocytic capacity, and the combined presence of hyperglycaemia and hyperinsulinaemia had an enhancing effect (if any) on neutrophil migration. While our studies were in progress, Fejfarova *et al.* also reported a lack of influence of short-term hyperglycaemia and/or hyperinsulinaemia on neutrophil phagocytic and respiratory burst capacity [42]. Together, these findings suggest that both chronic hyperglycaemia and/or hyperinsulinaemia impact on neutrophil functions and/or that other metabolic disturbances, such as dyslipidaemia or elevated levels of advanced glycation end-products, affect these functions.

Our study examined the acute effects of hyperglycaemia and/or hyperinsulinaemia and can therefore not directly be extrapolated to chronic conditions such as Type 2 diabetes and other insulin resistance syndromes. However, it is not feasible to study the chronic effects of isolated hyperglycaemia or isolated hyperinsulinaemia in healthy humans, in particular when using a tightly controlled design, such as implemented here. Instead, this study set-up has the exclusive ability to investigate two key components of Type 2 diabetes (hyperglycaemia and hyperinsulinaemia) separately from each other and from other metabolic disturbances that are part of Type 2 diabetes. We have shown that acute elevations in plasma glucose and/or insulin levels lead to alterations in an otherwise normally functioning immune system.

In conclusion, we have demonstrated that short-term hyperglycaemia reduced whereas combined hyperglycaemia and hyperinsulinaemia stimulated several, but not all, proinflammatory mRNA levels in blood leucocytes upon stimulation with LPS. Neither hyperglycaemia nor hyperinsulinaemia acutely affected cytokine concentrations or neutrophil functions.

## Abbreviations

C5a: complement 5a
EDTA: ethylenediamine tetraaceticacid
H_insu_E_gluc_: hyperinsulinaemic euglycaemic
H_insu_H_gluc_: hyperinsulinaemic hyperglycaemic
IκBα: inhibitor of kappa-Bα
IL: interleukin
L_insu_E_gluc_: lower insulinaemic euglycaemic
L_insu_H_gluc_: lower insulinaemic hyperglycaemic
LPS: lipopolysaccharide
mRNA: messenger RNA
NF-κB: nuclear factor kappa B
PAF: platelet-activating factor
PBS: phosphate-buffered saline
RFU: relative fluorescence units
TNF: tumour necrosis factor
